# Development of a pan-*Babesia* FRET-qPCR and a survey of livestock from five Caribbean islands

**DOI:** 10.1186/s12917-015-0560-0

**Published:** 2015-09-30

**Authors:** Jing Li, Patrick Kelly, Jilei Zhang, Chuanling Xu, Chengming Wang

**Affiliations:** Jiangsu Co-innovation Center for Prevention and Control of Important Animal Infectious Diseases and Zoonoses, Yangzhou University College of Animal Science and Technology, Yangzhou, Jiangsu 225009 P. R. China; Ross University School of Veterinary Medicine, Basseterre, St. Kitts and Nevis West Indies

**Keywords:** *Babesia* spp, FRET-qPCR, Livestock, Caribbean Islands

## Abstract

**Background:**

*Babesia* spp. are tick-borne protozoan hemoparasites and the second most common blood-borne parasites of mammals, in particular domestic animals. We used the Clustal Multiple Alignment program and 18S rRNA gene sequences of 22 *Babesia* species from GenBank to develop a PCR that could detect a wide variety of *Babesia* spp. in a single reaction. The pan-*Babesia* FRET-qPCR we developed reliably detected *B. gibsoni*, *B. canis*, *B. vogeli*, *B. microti*, *B. bovis*, and *B. divergens* under controlled conditions but did not react with closely related species, mainly *Hepatozoon americanum*, *Theileria equi*, and *Toxoplasma gondii*.

**Results:**

When we tested the pan-*Babesia* FRET-qPCR on DNA of whole blood from 752 cattle, sheep, goats, donkeys and horses from five Caribbean islands, we detected *Babesia* spp. expected to be present in the animals, mainly *B. bovis* and *B. bigemina* in cattle and *B. caballi* in horses and donkeys. Further, we found that animals were not uncommonly infected with species of *Babesia* usually associated with other hosts, mainly *B. vogeli* and *B. gibsoni* in cattle, sheep and goats, *B. rossi* in goats, and *B. caballi* in goats and sheep. Finally, the pan-*Babesia* FRET-qPCR enabled us to identify unknown species of *Babesia* in cattle, goats, sheep and donkeys.

**Conclusions:**

Overall, 70 % (525/752) of the animals we tested were positive confirming earlier limited studies that infections with *Babesia* spp. are common in livestock in the Caribbean.

## Background

*Babesia* spp. are tick-borne protozoan hemoparasites that occur worldwide [1–4]. They are the second most common blood-borne parasites of mammals, after trypanosomes, with infections occurring commonly in domestic animals, in particular cattle, dogs, horses, sheep, and pigs [5]. Recently, infections with *Babesia* (babesiosis) have been described in birds [6–8] and have attracted increasing attention as zoonotic infections in people [5, 9].

Since the first description of *Babesia* in cattle by Victor Babes in 1888, over 100 *Babesia* species have been identified [8]. Many cause significant economic losses in livestock, mainly *B. bovis* and *B. bigemina* in cattle [10], *B. motasi* and *B. ovis* in small ruminants [11] and *B. caballi* in horses and donkeys [12]. Further, *B. canis, B. vogeli and B. gibsoni* are important causes of morbidity and mortality in dogs worldwide [13] while *B. microti* and *B. divergens* are the species that most commonly infect people [8].

Initially, differentiation of the *Babesia* spp. was based on morphological and biological characteristics, and invertebrate and vertebrate host specificity. With the advent of molecular tools, however, these methods have proven to be of limited taxonomic value [8]. A number of nucleic acid-based techniques have been reported which detect *Babesia* spp. with high sensitivity and specificity. Most commonly, these assays have a narrow spectrum and specifically identify *B. microti*, *B. divergens*, or groups of *Babesia* spp. associated with specific hosts such as dogs [14, 15], cattle [16, 17] or sheep [18, 19]. To enable the detection of a wide range of *Babesia* spp. of veterinary and public health significance in a single reaction we developed a broad-based qPCR. Further, we tested our pan-*Babesia* FRET-qPCR on DNAs extracted from whole blood samples collected from five livestock species on five Caribbean islands. The results of these experiments are described below.

## Methods

### Whole blood

Whole blood samples (n = 752) were collected into EDTA from apparently healthy livestock on five Caribbean islands, including 162 from Dominica (cattle = 77, goats = 70, and sheep = 15), 31 from Grenada (all goats), 93 from Montserrat (cattle = 12, goats = 19, and sheep = 62), 198 from Nevis (cattle = 43, goats = 114, and sheep = 41) and 268 from St. Kitts (cattle = 193, goats = 4, sheep = 26, donkeys = 25, and horses = 20) [20]. The study was reviewed and approved by the Institutional Animal Care and Use Committee of the Ross University School of Veterinary Medicine (RUSVM), St Kitts.

### DNA extraction

After collection, the blood samples were transported on ice to RUSVM where red blood cells were separated by centrifugation and stored at −20 °C until thawed at room temperature and DNA extracted from aliquots (200 μL) using the QIAamp DNA Blood Mini Kit (QIAGEN, Valencia, CA, USA) according to the manufacturer’s instructions. The DNA was eluted in 200 μL washing buffer and shipped to Yangzhou University College of Veterinary Medicine of Jiangsu province, China at room temperature where it was frozen at −80 °C until PCRs were performed.

### Pan-*Babesia* FRET-qPCR

The PCRs in this study were performed on a Roche Light-Cycler 480-II platform. The HMBS-based quantitative PCR was used as an endogenous quality control to verify the quality of the DNA in the samples [21].

#### Primers and probes

**Fig. 1:**
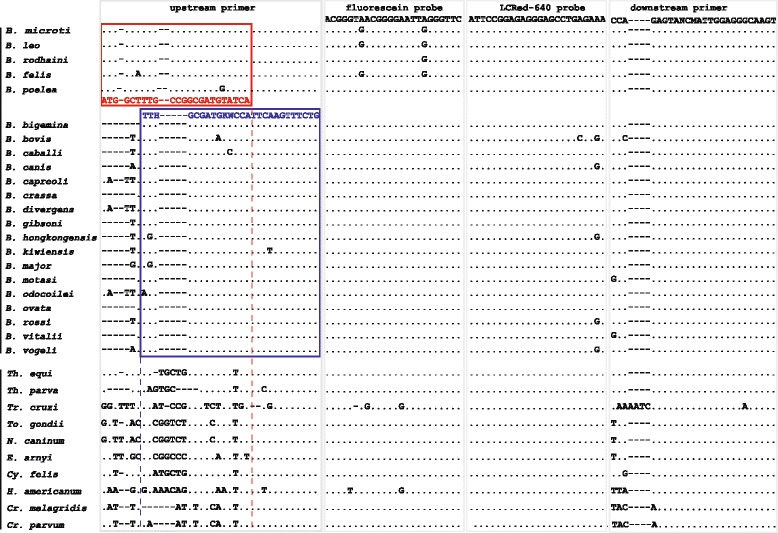
Alignment of the partial 18S rRNA gene amplicons of *Babesia* spp. and other related species. The upstream primer-1 (*in red*), upstream primer-2 (*in blue*), the fluorescein/LCRed 640 probes and the downstream primer are indicated in the top of the boxes. Dots indicate nucleotides identical to the primers and probes, and the dashes denote the deletion of the nucleotides. The upstream primers and two probes are used as the indicated sequences while the downstream primer is used as antisense oligonucleotide. While the probes and downstream primer show minimum mismatch with *Babesia* spp. and other related species, the upstream primers (−1 and −2) have 0–1 nucleotide mismatch with *Babesia* spp. but 6–16 nucleotide mismatches with the related non- *Babesia* species

The 18S rRNA sequences for 22 recognized *Babesia* spp. of public health significance and/or veterinary importance were obtained from GenBank (Fig. 1): *B. microti* (AB071177, AB219802), *B. leo* (AF244911, AY452708), *B. rodhaini* (DQ641423, AB049999), *B. felis* (AF244912, AY452707), *B. poelea* (DQ200887), *B. bigemina* (JQ723014, KF112076), *B. bovis* (HQ264124, HQ264127), *B. caballi* (AY534883), *B. canis* (AY072926, JN982353), *B. capreoli* (FJ944828, GQ304526), *B. crassa* (AY260176, JX542614), *B. divergens* (FJ944822, FJ944826), *B. gibsoni* (KJ142323), *B. hongkongensis* (JQ867356), *B. kiwiensis* (EF55315), *B. major* (JF802040), *B. motasi* (AY260179, AY533147), *B. odocoilei* (AY661508, U16369), *B. ovata* (AY081192, AY603400), *B. rossi* (JN982353), *B. vitalii* (JN880430, JN880431) and *B. vogeli* (HM590440). In addition, the 18S rRNA sequences of 9 related protozoan species were also obtained from GenBank: *Theileria equi* (AB515307, AB515312), *T. parva* (L02366), *Trypanosoma cruzi* (AF303659), *Toxoplasma gondii* (L37415), *Neosporo caninum* (U63069), *Eimeria arnyi* (AY613853), *Cytauxzoon felis* (AY679105), *Hepatozoon americanum* (AF176836), *Cryptosporidium meleagridis* (AF112574) and *C. parvum* (L16996).

These sequences were aligned using Clustal Multiple Alignment to identify conserved and variable regions suitable for primers and probes that could differentiate the species. The upstream primer-1 (5′-ATG GCT TTG CCG GCG ATG TAT CA-3′) was selected because of its high specificity for five *Babesia* species while a second upstream-2 primer (5′- TTT HGC GAT GKW CCA TTC AAG TTT CTG -3′) was selected because it reacted with 17 other *Babesia* species. Both had multiple mismatches with other related protozoan species (Fig. 1). The reverse primer (5′- CTG GCA CCA GAC TTG CCC TCC AAT -3′), the fluorescein probe [5′- ACG GGT AAC GGG GAA TTA GGG TTC-(6-FAM)-3′] and LCRed 640 probe [5′-(LCRed 640)-ATT CCG GAG AGG GAG CCT GAG AAA-PHOS-3′] were selected as they had only few mismatches amongst the *Babesia* but high numbers of mismatches with the related species we studied (Fig. 1).

#### Thermal cycling and melting curve analysis

The pan-*Babesia* FRET-qPCR was performed in a LighCycler 480-II real-time PCR platform using conditions described preciously [22] with a hybridization temperature of 58 °C. The PCR master mix contained the two upstream primers, the downstream primer, and two probes in concentrations described previously [23]. High-resolution melting curve analysis was performed following the completion of PCR [22]. Data were analyzed as 640 nm: 530 nm (F4/F1) fluorescence ratios, and the first derivative of F4/F1 (−d(F4/F1)/dt) was evaluated.

#### Sensitivity and specificity

To test the specificity of the pan-*Babesia* FRET-PCR, we used DNAs obtained in previous studies [20, 24] of *B. gibsoni*, *B. canis*, *B. vogeli* as positive controls and DNAs from *Hepatozoon americanum*, *Theileria equi* and *Toxoplasma gondii* as negative controls. In addition, we used plasmids created to contain the 18S rRNA gene (Integrated DNA Technologies, Coralville, IA, USA) of *B. microti*, *B. bovis*, and *B. divergens* as positive controls and *T. equi* as negative controls.

To test the sensitivity of the pan-*Babesia* FRET-PCR we used quantitative standards consisting of amplification products of PCRs for *B. gibsoni*, *B. canis* and *B. vogeli* identified in a previous study [14, 22]. The amplicons were gel purified with the QIAquick Gel Extraction Kit (Qiagen, Valencia, CA), quantified by the PicoGreen DNA fluorescence assay (Molecular Probes, Eugene, OR), and sequenced at the Genomic Sequencing Laboratory (GenScript, Nanjing, Jiangsu, China). The purified amplicons were diluted at 1,000, 100, 10, 1 genome copies per PCR reaction in T_10_E_0.1_ buffer as described previously [24], and used as quantitative standards.

In the specificity and sensitivity tests, the PCR products were electrophoresed through 1.5 % MetaPhor agarose gels. We calculated the size of the possible PCR amplicons for the different *Babesia* species to be between 282 to 293 bp and random samples with products within this range were purified for automated DNA sequencing (GenScript, Jiangsu, Nanjing, China) with a QIAquick PCR Purification Kit according to the manufacturer’s instructions (Qiagen, Valencia, CA, USA). The sequencing was performed by GenScript (Jiangsu, Nanjing, China) using the upstream and downstream primers to determine the *Babesia* species. In cases where there were poorly defined or multiple peaks in the sequencing results indicating mixed infections, pGEM®-T Easy Vector Systems (Promega, Madison, WI, USA) was used to clone PCR amplicons following the manufacturer’s instructions.

The 18S rRNA gene has been widely used to determine phylogenetic relationships between *Babesia* spp. [8] and it is generally accepted that the rRNA gene sequence similarity between two strains of the same species is over 98.65 % [25–27]. If the rRNA sequence for the *Babesia* isolate we obtained had a similarity of over 98.65 % with a *Babesia* species on GenBank, the isolate was regarded as being that species (Table 2).

### Confirmatory nested PCR

Nested primers (Outer primers: upstream: 5′-CATCAGCTTGACGGTAGGGTATT-3′, downstream 5′-CCCCCAACCGTTCCTATTAAC-3′; amplicon size: 489–518 bp; Inner primers: upstream: 5′-GAGGCAGCAACGGGTAACG-3′, downstream 5′-CCAACAAAATAGAACCAAAGTCCTA-3′; amplicon size: 421–447 bp) were designed to target a hyper-variable region of the 18S rRNA gene to amplify samples found positive for *Babesia* by FRET-PCR.

## Results

### Establishment of the pan-*Babesia* FRET-qPCR

The primers and probes we designed had 0–3 nucleotide mismatches with the 22 *Babesia* spp. with which they were compared, but 6–19 mismatches with 9 other related protozoan species (Fig. 1). The generic pan-*Babesia* FRET-qPCR we developed specifically detected DNAs of *B. gibsoni*, *B. canis*, *B. vogeli*, and plasmids from *B. microti*, *B. bovis*, *B. divergens*, but not DNAs from *H. americanum*, *T. equi* and *T. gondii*, and plasmids from *T. equi*. Using the purified *Babesia* DNAs (*B. canis*, *B. gibsoni* and *B. vogeli*) as quantitative standards, we determined that the detection limit of the FRET-qPCR was ~2 copies of 18S rRNA gene per PCR reaction, equivalent to 20 copies of 18S rRNA per ml whole blood. The *Babesia*-positive samples based on FRET-qPCR were verified in confirmatory nested PCRs and by sequence determination of the amplification products for precise species identification.

### Prevalence of *Babesia* spp. in Caribbean islands

Of the 752 blood samples we examined, 525 (70 %) were positive for *Babesia* spp. in our pan-*Babesia* FRET-qPCR. Although numbers were small, donkeys (88 %; 22/25) and horses (80 %; 16/20) were most commonly positive for *Babesia* spp. followed by cattle (78 %; 274/352), sheep (70 %; 101/144), and goats (47 %; 112/238). The results indicate *Babesia* spp. occur widely in the region with evidence of infection found on all islands studied, mainly Nevis (46 %; 92/198), Dominica (56 %; 90/162), Grenada (74 %; 23/31), and Montserrat (94 %; 87/93) St. Kitts (87 %; 233/268).

**Fig. 2:**
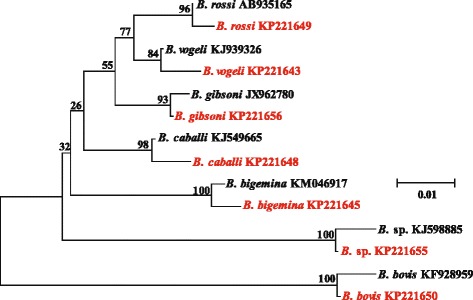
Phylogenetic comparison of the 282–293 bp variable region of the 18S rRNA gene found in the *Babesia* we found in our study (*in red*) and published *Babesia* sequences in GenBank (*in black*). Branch lengths are measured in nucleotide substitutions and numbers show branching percentages in bootstrap replicates

The PCR amplicons (282–293 bp) of our pan-*Babesia* FRET-qPCR were polymorphic among the different *Babesia* species and genomic sequencing thus enabled us to determine the species of *Babesia* amplified. Of the 525 *Babesia*-positive samples detected by the pan-*Babesia* FRET-qPCR, amplicons from 84 (84/525, 16 %) were randomly selected and sequenced revealing seven *Babesia* species were present in the livestock we studied from five Caribbean islands (Table 1, Fig. 2). These were *B. bigemina* (17/84, 20 %), *B. bovis* (6/83, 7 %), *B. caballi* (20/83, 24 %), *B. vogeli* (21/83, 25 %), *B. gibsoni* (8/83, 10 %), *B. rossi* (1/83, 1 %) and 11 unnamed *Babesia* spp. (11/83, 13 %).

**Table 1:** *Babesia* species identified by genomic sequencing in this study

Livestock	Island	Identified *Babesia* spp. by sequencing						
		*bigemina*	*bovis*	*caballi*	*vogeli*	*gibsoni*	*rossi*	*B.* sp.
Cattle	Dominica	6	1		6	1		1
	Montserrat	6	2		1			
	Nevis				7			
	St. Kitts	5	3		1			1
Goat	Dominica				2	1		
	Grenada			6				
	Montserrat				1		1	2
	Nevis							
	St. Kitts							
Sheep	Dominica							
	Montserrat			4	2			5
	Nevis							
	St. Kitts				1	5		1
Donkey	St. Kitts			5		1		1
Horse	St. Kitts			5

Cloning of PCR amplicons enabled us to identify dual infections with two or three *Babesia* species in 3 bovines from Montserrat (*B. vogeli* and *B. bigemina*), 1 bovines from Dominica (*B. vogeli* and *B. bigemina*), 1 goat from Montserrat (*B. vogeli* and *Babesia* sp.), 4 bovines from Dominica (*B. vogeli* and *B. gibsoni*), 2 bovines (*B. vogeli* and *B. bigemina*) and 1 sheep (*B. vogeli* and *B. gibsoni*) from St. Kitts, and 2 sheep from Montserrat (*B. vogeli*, *B. caballi* and an unnamed *Babesia* spp.).

While *B. caballi* was the main species identified by sequencing of positive amplicons from horses (5/5, 100 %) and donkeys (5/7, 71 %), up to four *Babesia* spp. were found in cattle, goats and sheep from the islands (Table 1). While DNA of *B. bigemina* and *B. bovis* were only found in cattle with the former being most prevalent (17/41, 41 %), DNA of *B. vogeli* and *B. gibsoni* was found in all the domestic ruminants. The DNA of *B. vogeli* was most commonly found in cattle (15/41, 37 %) while that of *B. gibsoni* was found most commonly found in sheep (5/18, 28 %). The DNA of *B. caballi* was not found in cattle but was found in sheep (4/18, 22 %) and also in goats (6/13, 46 %), where it was the most common species present. The DNA of *B. rossi* were found in only one goat while that of unnamed *Babesia* were found in all species on all islands but were most prevalent in sheep (6/18, 33 %; Table 1).

## Discussion

The pan-*Babesia* FRET-qPCR we developed proved to be both specific and sensitive in detecting *Babesia* spp. in controlled experiments. In all positive control reactions it identified *Babesia* spp. correctly and failed to give amplicons with the negative control organisms that were closely related to *Babesia* (Fig. 1)*.* The test could detect as few as 20 copies of the 18S rRNA gene per ml of whole blood indicating it would be useful in detecting chronic infections with low parasitemias that are common in infections with *Babesia* species. To the best of our knowledge, the pan-*Babesia* FRET-qPCR we designed is the first real-time PCR to be reported which detects multiple *Babesia* spp. with high sensitivity and specificity. We selected the 18S rRNA gene as the target for our FRET-qPCR as this is the most often used and reliable target gene for PCR detection of *Babesia* spp. [14]. Although the nucleotide sequences of the 18S rRNA gene are very similar amongst *Babesia* spp. and other related protozoan species, by systematically aligning the sequences of 32 *Babesia* and related species we were able to identify a highly conserved region specific for *Babesia* spp.. We developed specific primers (upstream primer-1 and upstream primer-2) to amplify this region and ensure that only *Babesia* spp. were detected and no related species. Further, the region of the 18S RNA gene we selected for our FRET-qPCR had nucleotide mismatches between the *Babesia* spp. we studied which enabled us to differentiate the organisms by sequencing the amplicons we obtained. When we compared the GenBank sequences of the 282–293 bp sections of the 18S rRNA gene of the recognized *Babesia* spp. detected by our pan-*Babesia* FRET-qPCR, we found each recognized *Babesia* sp. had 98.3 % or less similarity with the others (Table 2). This supports previous suggestions [25–27], based on comparisons of entire 18S rRNA gene sequences, that strains of organisms within the same species have similarities of over 98.65 %. When we applied our pan-*Babesia* FRET-qPCR to whole blood collected from five types of livestock from five Caribbean islands, we identified high prevalences of infections with *Babesia* species. This is not unexpected as the tick vectors of *Babesia* are common on livestock in the Caribbean [28, 29] and there are reports of high infection rates with *B. bigemina*, *B. bovis* and *B. caballi* [30–35]. The most prevalent *Babesia* species we found on cattle, *B. bovis* and *B. bigemina*, are transmitted by *Rhipicephalus microplus* which is very common in the Caribbean [29]. Although *R. microplus* has been found on sheep and goats in the Caribbean [28] and elsewhere [36] we found no evidence of transmission of *B. bigemina* or *B. bovis* in these small ruminants. We also found no evidence of the common small ruminant *Babesia* spp. mainly *B. ovis*, *B. motasi* and *B.* sp*.* Xinjiang [8] in the small ruminants we studied but this was not unexpected as their vectors, *Rhipicephalus bursa*, *Hemaphysalis* spp. and *H. longicornis*, respectively, do not occur in the region.

**Table 2:**
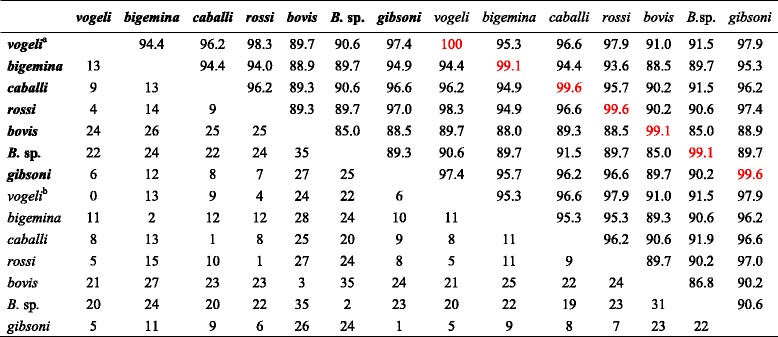
Percent similarities (upper-right diagonal half) and actual numbers of mismatches (lower-left diagonal half) in comparisons of 18S rRNA gene sequences of reference *Babesia* spp. (bold) and the isolates identified in this study (plain)

^a^The *Babesia* spp. in bold are recognized species from GenBank with Gene Accession numbers: KJ939326 for *B. vogeli*, KM046917 for *B. bigemina*, KJ549665 for *B. caballi*, AB935165 for *B. rossi*, KF928959 for *B. bovis*, KJ598885 for *B.* sp*.*, JX962780 for *B. gibsoni*

^b^The *Babesia* spp. in plain font are those we found on the Caribbean Islands. Their Gene Accession numbers are KP221643 for *B. vogeli*, KP221645 for *B. bigemina*, KP221648 for *B. caballi*, KP221649 for *B. rossi*, KP221650 for *B. bovis*, KP221655 for *B.* sp*.*, and KP221646 for *B. gibsoni*. The percent similarities between *Babesia* spp. above 98.65 % are shown in red

The commonest *Babesia* we found in the equids we studied was *B. caballi*, the agent of equine piroplasmosis which is common in the Caribbean [34]. There are at least 13 tick species incriminated in the transmission of *B. caballi* of which *Dermacentor* (*Anocentor*) *nitens* is considered to be the major vector in Latin America [37]. The tropical horse tick, *D. nitens*, is common on horses and donkeys in the Caribbean [28, 34] and can also be found on ruminants in the region [38] which might explain our findings of *B. caballi* in relatively high numbers of sheep and goats. Although we could find no reference to *B. caballi* infections in domestic ruminants, there is a report of a *Babesia* in desert bighorn sheep that had cross reacting antigens with *B. caballi* [39].

The traditional method of identifying *Babesia* species based on their size, numbers of daughter cells following merozoite division, and vertebrate and invertebrate host specificity have been replaced by molecular methods which have shown that *Babesia* have broader host ranges than thought previously [8]. This appeared to be the case in our study where we identified a number of *Babesia* not previously thought to infect livestock. The most common was *B. vogeli* which is a common cause of babesiosis in dogs around the world and also in the Caribbean [22, 40, 41]. Recently it has been reported in cats in Thailand [42] and lions in Zimbabwe [43], and our study extends its potential host range to domestic ruminants. The vector of *B. vogeli* is *Rhipicephalus sanguineus* which is found very commonly on dogs in the Caribbean [22, 40, 41]. While there are no reports of *R. sanguineus* on livestock in the Caribbean, the tick has been found occasionally on livestock elsewhere [44, 45]. Recently DNA of *B. vogeli* was found in *Rhipicephalus turanicus* in Israel [46] and this tick might be a vector of the organism. *Rhipicephalus turanicus* is morphologically very similar to *R. sanguineus* but is found on a wider host range including dogs, domestic ruminants and horses [47]. Further studies are indicated to determine if *R. turanicus* occurs in the Caribbean and if it is a vector of *B. vogeli*.

*Babesia gibsoni* is another agent of canine babesiosis which seems to be transmitted by the *R. sanguineus* group. The organism occurs widely, in northern Africa, southern Asia, Australia, Europe, the USA, the Caribbean and Central America [48], but ours is the first report of *B. gibsoni* in domestic ruminants. Similarly, although *Babesia rossi* was thought to be restricted to Africa where it is transmitted amongst dogs by *Haemaphysalis elliptica* [13], it has recently been reported in a *Haemaphysalis parva* in Turkey [49] and we now report the organism in a goat in the Caribbean. To the best of our knowledge, *Haemaphysalis* spp. have not been reported in the Caribbean and further studies are needed to determine the epidemiology of *B. rossi* in the area. As this organism is highly pathogenic in dogs it should be suspected in animals in the Caribbean that develop severe signs of canine babesiosis.

## Conclusions

In conclusion, our experiments have shown that the pan-*Babesia* FRET-qPCR we developed can reliably detect a variety of *Babesia* spp. under controlled conditions. Further, when used on bloods from livestock on Caribbean islands, it enabled us to detect *Babesia* spp. that were expected to be present in the animals we studied and to support previous studies showing infections are common in livestock in the Caribbean. In contrast to morphological studies and parasite specific PCRs, the pan-*Babesia* FRET-qPCR enabled us to demonstrated that livestock are not uncommonly infected with *Babesia* spp. that are usually associated with other hosts and that host specificity of *Babesia* spp. is likely less than thought previously.
